# Redox cofactors insertion in prokaryotic molybdoenzymes occurs via a conserved folding mechanism

**DOI:** 10.1038/srep37743

**Published:** 2016-11-25

**Authors:** Rodrigo Arias-Cartin, Pierre Ceccaldi, Barbara Schoepp-Cothenet, Klaudia Frick, Jean-Michel Blanc, Bruno Guigliarelli, Anne Walburger, Stéphane Grimaldi, Thorsten Friedrich, Véronique Receveur-Brechot, Axel Magalon

**Affiliations:** 1 Aix-Marseille Univ, CNRS, IMM, LCB UMR7283, Marseille, France; 2 Aix-Marseille Univ, CNRS, IMM, BIP UMR7281, Marseille, France; 3 Institut für Biochemie, Albert-Ludwigs-Universität, Freiburg, Germany; 4 Aix-Marseille Univ, CNRS, INSERM, Institut Paoli-Calmettes, CRCM UMR7258, Marseille, France

## Abstract

A major gap of knowledge in metalloproteins is the identity of the prefolded state of the protein before cofactor insertion. This holds for molybdoenzymes serving multiple purposes for life, especially in energy harvesting. This large group of prokaryotic enzymes allows for coordination of molybdenum or tungsten cofactors (Mo/W-*bis*PGD) and Fe/S clusters. Here we report the structural data on a cofactor-less enzyme, the nitrate reductase respiratory complex and characterize the conformational changes accompanying Mo/W-*bis*PGD and Fe/S cofactors insertion. Identified conformational changes are shown to be essential for recognition of the dedicated chaperone involved in cofactors insertion. A solvent-exposed salt bridge is shown to play a key role in enzyme folding after cofactors insertion. Furthermore, this salt bridge is shown to be strictly conserved within this prokaryotic molybdoenzyme family as deduced from a phylogenetic analysis issued from 3D structure-guided multiple sequence alignment. A biochemical analysis with a distantly-related member of the family, respiratory complex I, confirmed the critical importance of the salt bridge for folding. Overall, our results point to a conserved cofactors insertion mechanism within the Mo/W-*bis*PGD family.

The transition element molybdenum is essential for nearly all organisms and constitutes part of the catalytic center of a large variety of enzymes^1^,^2^. Importantly, molybdenum itself is catalytically inactive in enzymes unless it is complexed by an organic cofactor that is, with the exception of the one found in nitrogenase or carbon monoxide dehydrogenase, an ubiquitous pterin-based cofactor (i.e. Moco)^1^. The prokaryotic Mo/W-*bis*pyranopterin guanine dinucleotide (i.e. Mo/W-*bis*PGD) enzyme superfamily is one of the most prolific in the world of bioenergetics. The structural and catalytic module allowing coordination of the Mo/W-*bis*PGD cofactor and/or Fe/S clusters appears to have served multiple purposes in energy harvesting since the origin of life, and has consequently been termed “the catalytic workhorse of bioenergetics”^2^,^3^. Within the bacterial cell, successful synthesis and assembly of a molybdoenzyme is the result of a multitude of steps ranging from specific metal transport, Moco biosynthesis and trafficking, apo-target recognition, metal center insertion and, ultimately, acquisition of the intact properly folded and active conformation which can precede export in some cases^4^. Importantly, maturation culminates in the transfer of a bulky Moco to a structural protein recipient where it can span a distance of nearly 35 Å. The relevance of such a process must be seen in regards to the crucial role played by molybdoenzymes in human and microbial physiology^2^,^5^,^6^,^7^. However, a major gap in our knowledge of molybdoenzymes maturation is the identity of the prefolded state of the protein before Moco insertion. We addressed this question by determining the cofactor-driven conformational changes in one of the best-studied member of the Mo/W-*bis*PGD enzyme superfamily used as a model, the respiratory nitrate reductase.

Nitrate reductase A (NarGHI) found in most prokaryotes is involved in nitrate respiration under anoxic conditions^2^. This heterotrimeric and cytoplasmically-oriented complex is composed of (i) the nitrate-reducing subunit NarG containing a Mo-*bis*PGD (1541 Da) cofactor and a [4Fe-4S] cluster (FS0), (ii) the electron-transfer subunit NarH carrying four Fe/S clusters (FS1-4), and (iii) the membrane anchor subunit NarI containing two *b*-type hemes termed *b*_D_ and *b*_P_ according to their respective distal and proximal positions to the nitrate reducing site^8^. The dedicated chaperone NarJ plays a key role in the maturation process, allowing sequential insertion of FS0 and of Moco within the catalytic subunit NarG^9^. Furthermore, proofreading cofactor insertion through binding to a remnant Tat signal peptide at NarG, NarJ coordinates maturation and membrane targeting of the catalytic NarGH complex (~200 kDa)^10^,^11^,^12^. Importantly, multiple functions played by NarJ in the maturation process of the respiratory nitrate reductase complex can be extended to other members of the Mo/W-*bis*PGD superfamily (see for review^4^). The major challenge in defining the cofactor-driven conformational changes of a metalloprotein is to isolate and structurally characterize a stable prefolded state which remains competent for cofactor insertion. Remarkably, this is the situation encountered with the NarGH complex produced in absence of NarJ and devoid of both FS0 and Moco^9^,^13^. In such a system, we have chosen Small Angle X-ray Scattering (SAXS), a very powerful method in structural biology for the study of supramolecular assemblies, and which allows for comparative analysis of structural properties to identify conformational changes in solution.

We show here that the conformational changes associated with cofactors insertion are restricted to a structural motif of the Mo-*bis*PGD catalytic subunit. Phylogenetic analysis revealed a strict conservation of this motif within members of the Mo/W-*bis*PGD enzyme family. In particular, a solvent-exposed salt bridge of the catalytic subunit has a key role in enzyme folding after cofactors insertion as deduced from results obtained through site-directed mutagenesis, EPR spectroscopy and SAXS. Further, substitution of one of the residues involved in the salt bridge drastically reduces interaction with the dedicated chaperone. A biochemical analysis performed on the NADH:ubiquinone oxidoreductase (respiratory complex I), a related member of the family having lost the ability to coordinate the Moco while housing a Fe/S cluster equivalent to FS0, confirmed the key structural role of the salt bridge during enzyme maturation. Overall, our results point to a conserved cofactors insertion mechanism within the Mo/W-*bis*PGD family.

## Results

### SAXS analysis of an apo form of NarGH in solution

**Figure 1:**
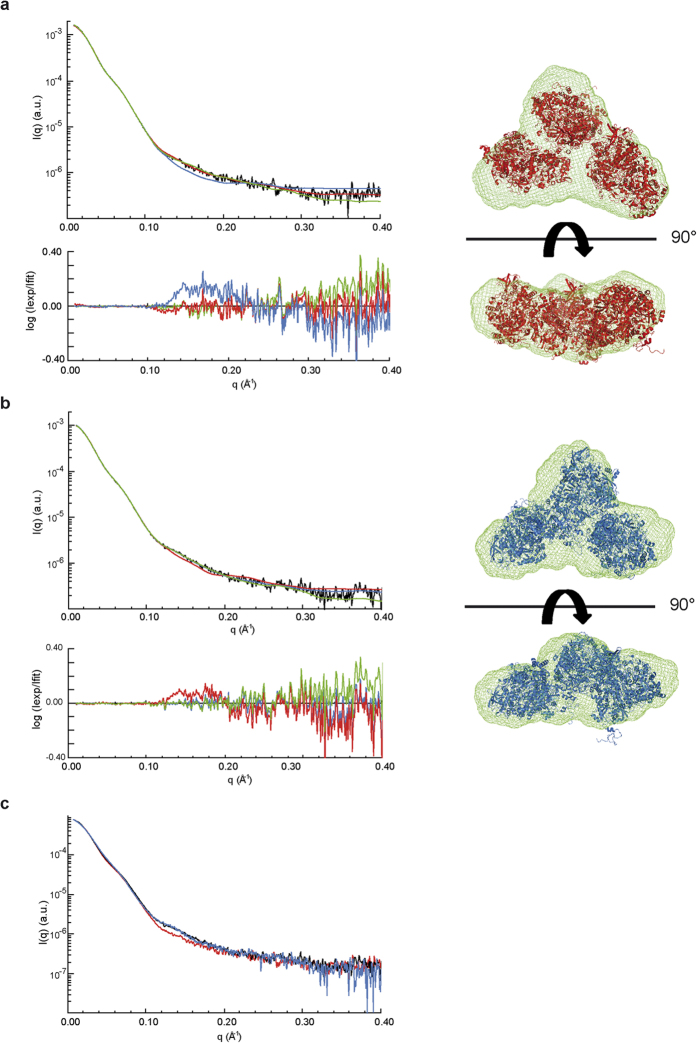
Conformational modifications of apo and holoNarGH revealed by SAXS rigid body modeling. (**a**) Left panel: experimental SAXS curve of holoNarGH (black), fits to the data by SASREF using 1q16 crystal structure (red, χ = 1.9) or 1q16 crystal structure with modification of the salt bridge between Arg108 and Glu794 (blue, χ = 2.3) and fit to the data by DAMMIF for *ab initio* shape determination (green, χ = 2.2); the corresponding residuals are plotted below; Right panel: Superimposition of structural organization of holoNarGH heterodimers (pdb code: 1q16) determined by SASREF (red) and of the overall shape determined *ab initio* by DAMMIF (green). (**b**) Left panel: experimental SAXS curve of apoNarGH (black) and fits to the data by SASREF (χ = 1.^5^ for 1q16 crystal structure and χ = 1.3 for the model with the modified salt bridge) and DAMMIF (χ = 2.1) using the same color code as for (A). The corresponding residuals are plotted below; Right panel: Superimposition of structural organization of apoNarGH (NarGH heterodimers issued from 1q16 with modified salt bridge) determined by SASREF (blue) and of the overall shape determined *ab initio* by DAMMIF (green). (**c**) Superimposition of the experimental SAXS curves of holoNarGH (red), apoNarGH (black) and NarG_R108A_H variant (blue).

To evaluate the conformational changes associated with Moco insertion into the soluble catalytic NarGH complex, SAXS was performed on both an apo form as obtained in absence of NarJ (i.e. apoNarGH) and on the Moco-loaded form (i.e. holoNarGH). Guinier analysis and determination of the distance distribution function revealed that the two proteins have similar dimensions, with a radius of gyration (R_g_) of 63.1 ± 0.3 and 63.2 ± 1.1 Å and maximum dimension D_max_ of 200 and 210 Å for holoNarGH and apoNarGH, respectively. Direct determination of the molecular weight (M_w_) from the scattering intensities using the program SAXSMoW^14^ led to values of 580 ± 60 kDa and 540 ± 55 kDa for holoNarGH and apoNarGH respectively, to be compared to the M_w_ of 200 kDa for NarGH based on the primary structure. These values and associated error bars inferred from the scattering intensities (i.e. independent of the overall shape of the protein) reveal that holoNarGH and apoNarGH samples analyzed by SAXS are both trimeric (NarGH)_3_ in solution. Identical conclusions were made using size-exclusion chromatography supporting the oligomeric state of the fractions subsequently analyzed by SAXS (Supplementary Fig. 1). *Ab initio* shape restoration of the two proteins produced trilobal shapes that can accommodate three NarGH heterodimer atomic structures, with very good fit to the data (Fig. 1a and b). Indeed, the calculated volume of these envelopes (809,600 Å^3^ and 765,300 Å^3^ for holo and apoNarGH, respectively) corresponds to three times the volume of the NarGH crystal structure (247,600 Å^3^), further confirming the trimeric organization of both holo and apoNarGH in solution.

**Figure 2:**
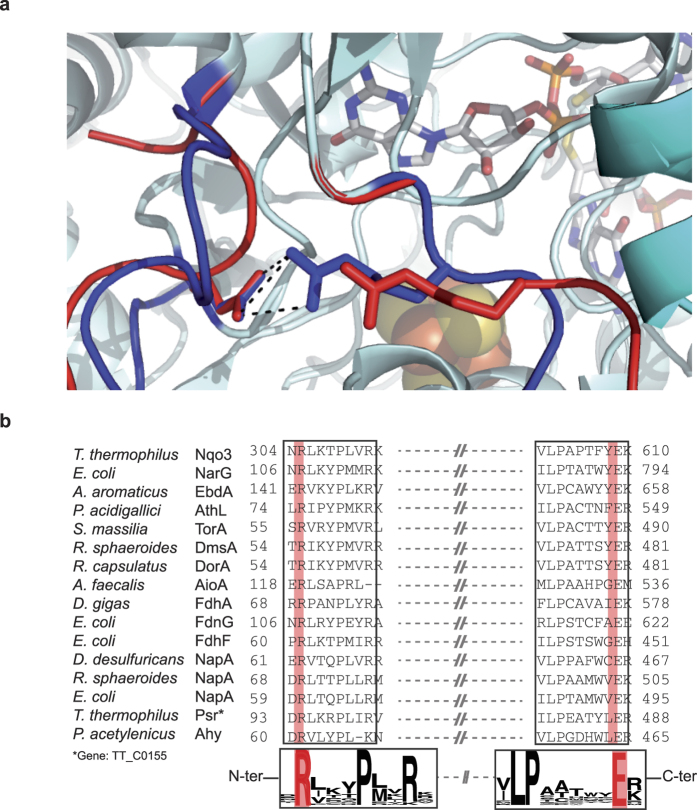
A solvent-exposed salt bridge is structurally and phylogenetically conserved in catalytic subunits of Mo/W-*bis*PGD enzyme family. (**a**) Close-up view of the solvent-exposed salt bridge (NarG_R108_-NarG_E794_) within the NarGH catalytic dimer. Secondary structure of NarG is colored in cyan. The NarG_R108_ and NarG_E794_ are represented in sticks as well as the Mo-*bis*PGD cofactor present in NarG. The Fe/S cluster in NarG is shown in sphere. The loops bearing the Arg108 and Glu794 residues are colored in blue in holoNarGH (pdb 1q16) and colored in red in the model structure of apoNarGH. (**b**) 3D structure-guided multiple sequence alignment of selected representatives of Mo-*bis*PGD-harboring catalytic subunits completed with close-related NuoG sequences. Details concerning the selected sequences are found in online methods.

Interestingly, the two proteins exhibit different conformations, as attested by differences in the SAXS profiles, especially in the mid-q region (0.10–0.20 Å^−1^, see Fig. 1c). To gain insight into these cofactor-dependent conformational changes, rigid body modeling was performed using the SASREF program and different atomic models of NarGH. Using the atomic coordinates from the X-ray crystal structure of NarGH (Protein data Bank code 1Q16), an excellent fit was obtained for the holoNarGH SAXS curve (Fig. 1a, red trace versus black trace), giving rise to a global shape similar to the trilobal one calculated *ab initio* by DAMMIF (Fig. 1a, green trace). On the contrary, the conformation adopted by apoNarGH differs from that of holoNarGH visualized in the X-ray crystal structure (Fig. 1b, red trace versus black trace). Examination of the global shape envelope of apoNarGH as calculated by DAMMIF points out towards the NarG subunit as being the site for most conformational changes. Rigid body modeling was thus performed to generate hypotheses about the spatial arrangement of the NarG subunit within the apoNarGH complex (see methods). The strategy used for modeling was based on the assumption that cofactor-dependent conformational changes should allow insertion of the bulky Moco molecule (1541 Da) at the interior of the NarG subunit within the apoNarGH complex. First, the flexibility of domain IV of NarG (residues Lys1078 to Ile1184) (Supplementary Fig. 2) was assessed as it was shown to be mobile in a related member of the Mo/W-*bis*PGD family, and proposed to serve as a lid after cofactor insertion^15^. The domain IV of NarG was given as an independent structure in SASREF allowing free movement with respect to the rest of NarGH with only distance restraints of 4 Å maximum between Gln1077 and Lys1078 and also between Ile1184 and His1185 of NarG, consistent with formation of peptide bonds between these residues. During the minimization process, several positions of domain IV were tested, but the best fit to the apoNarGH SAXS data was obtained with domain IV at the same position as in the holoNarGH complex, thereby conferring no improvement of the fit to apoNarGH SAXS data compared to holoNarGH. This indicates that domain IV of NarG is not mobile (at least upon detection by SAXS) and cannot account for the structural changes observed between apo and holoNarGH (Fig. 1c). Second, the guanine moiety of the Moco molecule is protected from the solvent by loops connected by a surface-exposed salt bridge at a distance of less than 10 Å. We noticed that disruption of this salt bridge between Arg108 and Glu794 in NarG would modify the surface of NarGH so that it allows access to the cofactor pocket (Fig. 2a and Supplementary Fig. 3). Modification of the loops was performed and allowed for the use of the atomic model thus generated as a template for SASREF. Using this model, the fit to the SAXS data of apoNarGH was significantly improved, especially in the 0.10–0.20 Å^−1^ q-range (Fig. 1b, blue trace versus black trace). Concomitantly, this model gave rise to a poorer fit with the holoNarGH data than the one obtained with the crystal structure (Fig. 1a, blue trace versus black trace). Overall, our results indicate that disruption of a solvent-exposed salt bridge in NarG can account for the observed conformational changes occurring in apoNarGH.

### The salt bridge is strictly conserved into the Mo/W-*bis*PGD enzyme family

The structural and catalytic subunit of the Mo/W-*bis*PGD enzyme family is conserved in a large number of prokaryotic enzymes such as formate dehydrogenase (Fdh), nitrate reductases (Nar, Nap), arsenite oxidase (Aio) or polysulfide reductase (Psr). The clear-cut cleavage between archaeal and bacterial protein sequences in the Nar, Fdh, Aio and Psr clades suggests this subunit housing a Moco and a Fe/S cluster to have been present in the Last Universal Common Ancestor (Supplementary Fig. 4). Thereof, phylogenetic calculations suggest that the Mo/W-*bis*PGD structural subunit has served multiple purposes in energy harvesting at the origin of life^16^. Interestingly, significant similarity between the C-terminal sequence of NuoG subunit of respiratory complex I from bacteria and eukaryotes and the catalytic subunit of Mo/W-*bis*PGD enzyme family has been noted^17^,^18^,^19^. Bacterial complex I is generally made up of 14 subunits called NuoA-N (for NADH:ubiquinone oxidoreductase). Subunits NuoB, E, F, G and I carry Fe/S clusters ensuring electron transfer between NADH and the substrate quinone. NuoF carries the NADH binding site. NuoG contains one [2Fe-2S] and three [4Fe-4S] clusters but is devoid of Moco. One of the [4Fe-4S] clusters called N7 is not strictly conserved amongst all species but is located at a homologous position in the C-terminal region of NuoG with respect to FS0 in NarG (Supplementary Fig. 3). N7 is not involved in the electron transfer reaction but is essential for the stability of the complex^20^. The resolution of the crystal structure of complex I from *Thermus thermophilus*^21^ enables refinement of the multiple sequence alignment of NuoG homologues with members from this superfamily (Fig. 2b) and reveals that the arginine and glutamate residues involved in the formation of the salt bridge in NarG are strictly conserved in members of the Mo/W-*bis*PGD enzyme family. This makes NuoG as a unique example of the family having lost the ability to hold Moco during evolution while conserving an equivalent Fe/S cluster and the surface-exposed salt bridge (Supplementary Fig. 3).

### The R108-E794 salt bridge is essential for NarGH folding once redox cofactors have been inserted

**Figure 3:**
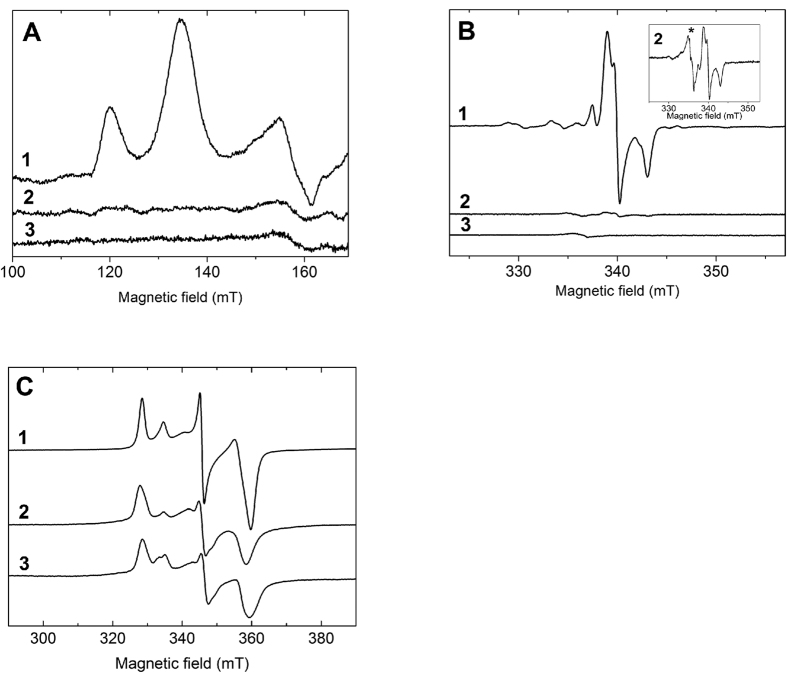
The NarG_R108A_ substitution impacts nitrate reductase cofactor content. EPR spectra of the FS0 [4Fe-4S]^1+^ (**A**), Mo^V^ (**B**) and FS1 [4Fe-4S]^1+^ (**C**) cofactors in redox-poised purified samples containing holoNarGH (1), NarG_R108A_H (2), and apoNarGH (3). The redox potentials at which the samples were poised are: (**A**) −425 mV (1), −436 mV (2) and −409 mV (3); (**B**) +178 mV (1), +171 mV (2) and +198 mV (3); (**C**) −149 mV (1), −175 mV (2) and −161 mV (3). Experimental conditions were: microwave frequency,~9.41 GHz; temperature, 9 K (**A**), 50 K (**B**) or 12.5 K (**C**); microwave power, 100 mW (**A,C**) or 4 mW (**B**); field modulation amplitude, 1 mT (**A**), 0.4 mT (**B**) or 0.5 mT (**C**) at 100 kHz, using a single scan. The inset in (**B**) shows the Mo^V^ spectrum in the NarG_R108A_ complex after 25 accumulations. A minor radical signal indicated by an asterisk in the inset was detected in both NarG_R108A_H and apoNarGH samples.

**Figure 4:**
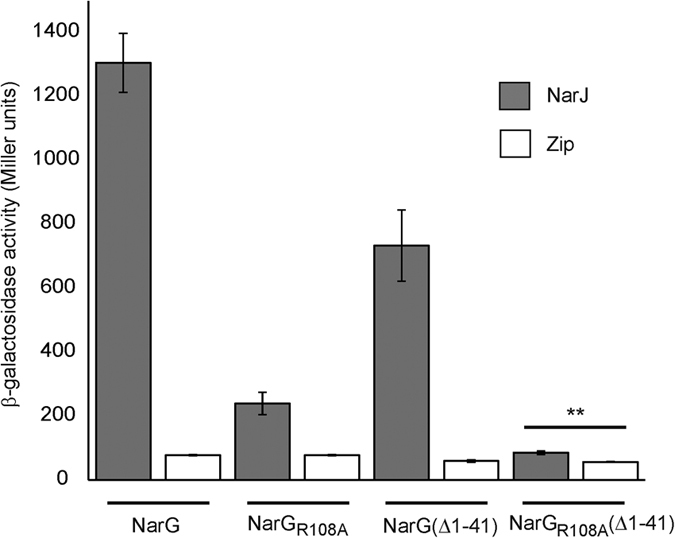
The NarG_R108A_ substitution severely impairs interaction with the dedicated chaperone NarJ. Interactions have been measured between NarJ, NarG, NarG(Δ1-41) and their corresponding variants. Interaction with the Zip domain was used as a negative control. The β-galactosidase activity values are the average of at least three independent experiments and are expressed in Miller units. A statistical test was performed by the no parametric Wilcoxon/Mann–Whitney method. Two stars stand up for a p value ≤0.02.

To confirm the importance of the salt bridge during NarGH maturation, site directed mutagenesis was used to substitute the Arg108 residue by an Ala and the variant was purified. The variant showed a strongly reduced benzyl-viologen:nitrate oxidoreductase activity (5 units/mg of nitrate reductase) as compared to holoNarGH (90 units/mg). Metal quantitation by ICP-MS indicated a lower Fe content in the variant (15 ± 2 Fe/NarGH) than in holoNarGH (19 ± 2 Fe/NarGH) and less than 10% of Mo, demonstrating a significant alteration of the metal cofactor content in NarG_R108A_H. To deeper evaluate the molecular consequences of the disruption of the salt bridge on the NarGH cofactor content, a comparative EPR spectroscopy analysis of the NarG_R108A_H complex was carried out. The EPR signature of the NarH [3Fe-4S]^1+^ cluster FS4 was identical in the oxidized state of the variant and wild-type enzymes, indicating that this cluster is not affected by the substitution. In contrast, in reduced states, the two low-field resonances at g ~ 5.6 and 5.0 characteristic of the S = 3/2 ground state of the NarG [4Fe-4S]^1+^ FS0 cluster^22^,^23^ were absent in the NarG_R108A_H complex (Fig. 3A). Examination of EPR spectra around g = 2 of redox poised NarG_R108A_H indicated that, by comparison with wild-type enzyme, no additional EPR signature was detected, which likely excludes any conversion of the FS0 spin state into a lower S = ½ spin state in the investigated [+400, −450] mV redox potential range. Interestingly, similar changes have been reported in apoNarGH complex produced in the absence of the NarJ chaperone (Fig. 3A)^9^. However, whereas the absence of NarJ also precludes the insertion of Moco into NarG, a weak but unchanged Mo^V^ EPR signal was detected in the NarG_R108A_H complex (Fig. 3B). Spin quantitation indicated that its relative proportion to FS4 (~1%) was strongly decreased in comparison to that typically measured in holoNarGH (i.e. ~30–40%). In addition, alteration of the composite EPR signature of the FS1 cluster was observed in the NarG_R108A_H complex (Fig. 3C). The quasi-axial component that forms about 33% of the composite FS1 EPR signature in holoNarGH^24^,^25^ was not detected in the substituted enzyme and the EPR lines associated to the remaining rhombic component were broadened (Fig. 3C). Such a broadening arises from g-strain effect enhancements reflecting increased flexibility of the FS1 environment in the variant enzyme. This flexibility is in line with the tolerance of the FS1 environment to structural constrain variations supported by the ability to selectively remove this cluster without affecting the properties of the other FeS clusters from NarH^9^,^26^. A similar spectrum was recorded on apoNarGH (Fig. 3C). These results indicate that disruption of the salt bridge induces a structural perturbation of the FS1 cluster, which is similar to the one that occurs when NarGH is produced without NarJ. It is worth to recall that the edge-to-edge distance between FS1 and the Mo-*bis*PGD cofactor is only 12.2 Å. In addition to these observations, the solution structure of NarG_R108A_H was evaluated by SAXS (Fig. 1c). The dimensions of NarG_R108A_H (R_g_ = 63.6 ± 0.9 Å and D_max_ = 209 Å) and inferred M_w_ using SAXSMoW (590 ± 50 kDa) were identical to holo and apoNarGH within the error bars. Strikingly, the scattering profiles of the variant and of apoNarGH were indistinguishable, indicating that the two complexes have the same conformation in solution. These results validate the absence of the salt bridge in the apoNarGH complex thereby in an open conformation facilitating cofactors insertion. Our hypothesis is that these cofactor-dependent conformational changes at the protein surface are sufficient to allow NarJ recognition thus initiating cofactors insertion. We reasoned we could test this hypothesis by evaluating NarJ binding to the R108A variant. To do this, we performed two-hydrid experiments as previously^10^. As seen in Fig. 4, interaction between the R108A NarG variant and NarJ is severely affected even in the absence of the remnant Tat signal peptide known to be one of the two NarJ binding sites onto NarG^10^. These data provide an explanation for the lower cofactors content of the NarGH variant and the similar open conformation to apoNarGH produced in absence of NarJ. Overall, integrity of the R108-E794 salt bridge is essential for production of a fully mature and active NarGH complex.

### The conserved salt bridge in the related NuoG subunit is important for complex I stability

**Figure 5:**
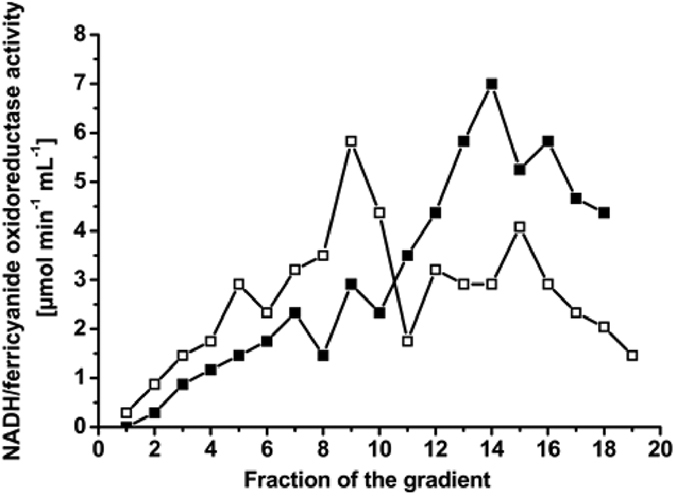
The conserved salt bridge in the related NuoG subunit is important for complex I stability. Activity of sucrose gradient fractions of detergent extracts from cytoplasmic membranes of strains BW25113Δ*ndh*Δ*nuo*/pBAD*nuo*_*his*_ (■) and BW25113Δ*ndh*Δ*nuo*/pBAD*nuo*_*his*_ *nuoG* E615A (□). Activities were normalized for a load of 10 mg protein per gradient to allow direct comparison. Fractions are numbered from top (1) to bottom (20).

We next questioned the role of the conserved salt bridge for folding of the unique member of the Mo/W-*bis*PGD family, NuoG which has lost the ability to bind Moco during evolution while housing a Fe/S cluster equivalent to FS0, named N7 cluster. The Glu615 residue of NuoG (equivalent to Glu794 in NarG) was substituted into Ala and the impact on activity and structural integrity of complex I was evaluated (Supplementary Fig. 3). Experiments being performed in a Δ*ndh* strain, lacking the alternative NADH dehydrogenase, all NADH-related activities stem solely from complex I. NADH:ferricyanide oxidoreductase and NADH oxidase activities measure the amount of complex I in the membrane and its catalytic activity, respectively. Both activities were reduced by one third in the variant (1.0 ± 0.1 and 0.14 ± 0.02 units versus 1.4 ± 0.1 and 0.21 ± 0.02 units for the variant and the wild-type, respectively). The artificial NADH:ferricyanide oxidoreductase activity showed that the amount of the complex in the mutant membranes is about two thirds of that of the parental strain indicating a slight effect of the mutation on the assembly of the complex. However, normalization of the NADH oxidase activity with the amount of complex in membranes as evaluated by western-blot analysis on NuoF (Supplementary Fig. 5) confirmed that the mutation has no impact on the specific activity (0.2 μmoles. min^−1^. mg^−1^). The full activity of the assembled and correctly located variant complex indicates that it contains all the cofactors, namely one flavin mononucleotide and eight Fe/S clusters that are indispensable for its activity. However, *E. coli* complex I contains one more Fe/S cluster, N7 that is not involved in the activity and that is located in NuoG. Next, stability of complex I was assessed by sucrose gradient centrifugation of a detergent-extract of membranes containing the variant or wild-type complex. The presence of a fully assembled and stable complex I is indicated by an activity peak at two-thirds of the gradient (fractions 12 to 15) as shown for the wild-type (Fig. 5). In contrast, the activity peak of the variant sedimented in a fraction of lower density, indicative of the presence of the soluble NADH dehydrogenase fragment of the complex made up of the NuoEFG subunits^27^. The residual Nuo subunits comprise another fragment of the complex that is still bound to the membrane but does not exhibit NADH:ferricyanide oxidoreductase activity and thus is not detected by our methods^20^. Furthermore, we were not able to purify further the NuoEFG variant subcomplex by chromatographic steps due to its strongly reduced stability once solubilized from the membrane. These findings are indicative for the loss of Fe/S cluster N7 as described earlier^20^. Because N7 is not involved in the electron transfer reaction, its loss has no influence on the physiological activity of the complex. In summary, the loss of the salt bridge leads to effects that can be fully explained by the loss of cluster N7 accompanying conformational changes.

## Discussion

In this study, the overall structure of the cofactor-less NarGH complex from *E. coli* has been determined by SAXS and allowed for identification of cofactor-dependent conformational changes. Most importantly, our results point towards a conserved cofactors insertion mechanism in enzymes displaying the Mo/W-*bis*PGD structural module.

The biogenesis of members of the prokaryotic Mo/W-*bis*PGD enzyme family including the nitrate reductase complex is a particularly involved process because of the complexity and oxygen-sensitivity of the Moco molecule (see for review^4^,^28^). Within this family, Mo/W-*bis*PGD insertion involves a biosynthetic/delivery platform formed by an extensive network of interactions among proteins involved in the final stages of Moco biosynthesis^29^. The current view is that such a biosynthetic platform ensures both the fast and protected transfer of reactive and oxygen-sensitive intermediates during the reaction sequence of Moco biosynthesis^30^,^31^. Moco insertion is often assisted by dedicated chaperones ensuring coordination of folding and cofactor insertion events^4^,^32^. In *E. coli* nitrate reductase A (NarGHI), the dedicated chaperone NarJ is a crucial component of the Moco insertion process in authorizing the interaction of apoNarGH with the Moco biosynthetic/delivery platform^33^. Importantly, NarJ binding is also responsible for insertion of the proximal Fe/S cluster FS0 and NarJ-assisted FS0 insertion constitutes a prerequisite for Moco insertion^9^. While the exact function of NarJ in this process is unclear, FS0 insertion likely induces subtle conformational changes at the interior of NarG such as stabilizing the ^49^CGVNCTG^55^ sequence (including two cysteine ligands of the FS0 cluster) facilitating subsequent Moco insertion in close proximity^34^. This sequence is further involved in a number of interactions with the pyranopterin and guanine moieties of Moco. Such sequence of events for cofactors insertion could be a more general feature of this enzyme family as a similar situation has been since recognized for the related *E. coli* DMSO reductase^35^. Despite extensive studies on several members of the Mo/W-*bis*PGD enzyme family emphasizing common rules governing their folding process^4^,^32^,^36^, there is a lack of knowledge on the prefolded state of the catalytic subunit which is amenable for interaction with cofactor biosynthetic/delivery platforms which may include the participation of a dedicated chaperone.

Although highly variable in size and sequence (from 710 to 1200 residues) and resulting from a long evolution from the origins of life, all the known structures of members of the Mo/W-*bis*PGD enzyme family share a common core protein fold divided into four different domains (I-IV) that bury the cofactors^16^. The archetypal subunit contains a Fe/S cluster known as FS0 in addition to the Mo/W-*bis*PGD herein exemplified by NarG. Several deviations from this archetype are found during evolution such as the loss of the Fe/S cluster and/or Fe/S-rich N- or C-terminal extensions. Such a situation is encountered with NuoG used herein as a more distantly-related member housing solely a Fe/S cluster. Resolution of the crystal structure of complex I from *Thermus thermophilus*^37^ allows to dissect NuoG into two separate domains: a N-terminal one homologous to [NiFe] hydrogenases and containing three Fe/S clusters, and a C-terminal one homologous to members of the Mo/W-*bis*PGD enzyme family containing the N7 cluster in the equivalent position to FS0 and an empty Moco binding site^17^,^18^,^19^. The overall RMSD value of this C-terminal domain with NarG is 2.962 Å, both sharing a strikingly similar overall fold including the loops involved in the formation of the surface-exposed salt bridge (Supplementary Fig. 3). The Mo-*bis*PGD cofactor is comprised of two antiparallel pyranopterins with a twofold axis of symmetry through the Mo. As such, domains II and III bind the guanine moieties of each pterin which extend close to the protein surface. At one extremity of the Moco molecule, domain IV constitutes a cap whose rotation would allow access to a wide funnel for Moco insertion as recently suggested^15^,^28^. Notably, our comparative SAXS analysis of both the apoNarGH and holoNarGH revealed that cofactor-dependent conformational changes are restricted to the catalytic subunit NarG. Mobility of domain IV was specifically addressed to account for the observed structural differences in the apoNarGH complex. However, rigid body modeling analysis by SASREF ruled out this hypothesis. At the other extremity of the Moco molecule, the guanine moiety is protected from the solvent through the formation of a surface-exposed salt bridge between Arg108 and Glu794 residues present in flexible loops of domain I at a distance of nearly 10 Å. Three lines of evidence support the finding that an open conformation as obtained in absence of the salt bridge allows direct access from the outer surface to a wide funnel including the FS0 binding motif and that this prefolded state is competent for cofactors insertion. First, corresponding modification of the X-ray atomic model of holoNarGH significantly improved the fit with the apoNarGH SAXS data. Further, SAXS analyses demonstrate that disruption of the salt bridge mimics the conformation adopted by the apoNarGH complex produced in absence of NarJ. Second, previous biochemical and spectroscopic experiments support that cofactors insertion proceeds in absence of the salt bridge. Effective *in vitro* cofactor insertion in apoNarGH (with or without FS0) and restoration of an active state have been reported by us^10^,^13^. Third, disruption of the salt bridge by the R108A substitution severely impairs interaction with NarJ, key for cofactors insertion. The implications of these findings are that low activity and metal content in the NarGH variant result from a defective cofactors insertion process. The open conformation of the variant may additionally affect stability of FS0 located at nearly 15 Å from the surface and leads to its subsequent loss. Furthermore, our data support the view that Arg108 is involved in NarJ interaction in the open conformation of the NarGH complex. The conservation of the arginine residue points towards a conserved mode of folding within members of the Mo/W-*bis*PGD family. Conversely, closure of the salt bridge as deduced from X-ray crystal structure analysis of the Moco-free complex^22^ prevents further Moco insertion using *in vitro* assays^10^. Indeed, this form of the enzyme complex has acquired a definitive conformation likely through acquisition of FS0 and a guanine nucleotide proximal to the salt bridge and at the corresponding position of the Mo-*bis*PGD molecule in the native complex. Such observation suggests that the salt bridge may form even without Moco insertion if sufficient folding is attained by cofactors insertion, herein FS0 and a GDP.

Interestingly, during the course of evolution, complex I subunits have been diversely adapted to meet physiological and energetic needs in prokaryotes as recently disclosed by phylogenomic studies^38^. In particular, the Mo/W-*bis*PGD related NuoG subunit of complex I has been subjected to several modifications such as a varying number of Fe/S clusters (from three to five) and the loss of the ability to bind Moco^18^,^39^,^40^,^41^. Such plastic rearrangement is likely due to the prominent position of NuoG within the multiprotein complex close to the catalytic site. Importantly, the residues involved in the salt bridge are conserved in complex I from prokaryotes to eukaryotes. This unique example allows to question the role of the salt bridge for folding of a Moco-less domain. As illustrated here with *E. coli*, our results clearly show that disruption of the conserved salt bridge in NuoG leads to the formation of a fully active assembled and correctly located complex while severely destabilized upon membrane solubilization. Such a situation is reminiscent of NuoG variants having lost the ability to coordinate the N7 cluster^20^. It is worth mentioning that assembly of complex I proceeds in a stepwise manner with the final membrane association of a catalytic module made up of the NuoEFG subunits. Salt bridge disruption likely impacts the occupancy of the proximal N7 cluster by reducing insertion or maintenance and further precludes complete folding of the domain. Since N7 plays only a structural role and is not involved in electron transfer, its loss resulting from salt bridge disruption or coordinating cysteine variants^20^ has no impact on the activity but a detrimental effect on its overall stability. Overall, during evolution the surface-exposed salt bridge has kept its role of folding during the course of redox cofactor acquisition being either a Fe/S cluster followed by Moco in Nar or simply a Fe/S cluster in Nuo.

Not much is known concerning the prefolded state of other molybdoenzymes still competent for cofactors insertion. Whilst preliminary, a SAXS study performed on a chaperone-molybdoenzyme complex, apoTorAD showed a largely folded catalytic subunit^15^. Herein, Moco is the sole prosthetic group of TorA, catalytic subunit of the trimethylamine *N*-oxide reductase and member of the Mo/W-*bis*PGD family^42^. TorD represents the dedicated chaperone for TorA in charge of Moco insertion^43^,^44^,^45^. The authors showed that the apoTorAD complex adopts the same overall conformation even in absence of the Tat signal peptide, known to constitute one of the TorD-binding sites^45^. Moreover, TorD is likely positioned opposite to the domain IV of TorA further excluding movement of domain IV as being the site for TorD-driven Moco insertion. Further structural investigation are required to evaluate the opening of the structure allowing Moco insertion as well as the role of the conserved salt bridge for TorD interaction. Cofactor insertion in the case of members of the sulfite oxidase family appears to be straightforward, as Moco is not deeply buried in the holoenzyme. However, the first insight for the cofactor insertion process into sulfite oxidase has only been provided recently through the use of a site-directed spin-labeling strategy^46^. The authors observed conformational changes at the protein surface which may allow access to the protein interior. Contrarily, Moco insertion into members of the xanthine oxidase family appears more challenging owing to its buried position. A current working model by Hille^47^ is based on the motion of a highly conserved structural motif at the interface of the catalytic subunit dimer providing access to both Moco binding pockets. Such motif may also be important for recruiting the dedicated chaperone XdhC in charge of Moco insertion^48^. While these two systems are structurally unrelated to members of the Mo/W-*bis*PGD family, a common trait is the partial opening of an already largely folded protein which, in some cases, already contains cofactors.

In light of our findings, we propose the following model for how cofactors insertion operates *in vivo* into the catalytic subunit of the nitrate reductase complex. In addition to recruiting the Moco biosynthetic/delivery platform, formation of the complex between the dedicated chaperone NarJ and the apoenzyme likely maintains an open conformation of the catalytic subunit and allows sequential insertion of FS0 and Moco. In particular, FS0 insertion would stabilize internal loops harboring coordinating cysteines, thus facilitating subsequent Moco insertion. Our studies suggest that the R108 residue present on a flexible loop is involved in NarJ interaction supporting its proximity to the cavity at which cofactors insertion proceeds. After insertion of both cofactors, formation of the salt bridge terminates the folding process and likely resolves the holoenzyme-chaperone complex. The strict conservation of the residues involved in the surface-exposed salt bridge in all members displaying the Mo/W-*bis*PGD structural module suggests that the observed conformational changes in the nitrate reductase complex during the folding process and the proposed redox cofactors insertion mechanism apply to other members within the family as well.

## Methods

### Bacterial strains and plasmids

The *E. coli* strains and plasmids are described in Supplementary Table 1. Derivatives from *E. coli* strain BW25113^49^ were used for overproduction of complex I and the variant. As such, strain BW25113Δ*nuo* lacks the chromosomal *nuo*-operon encoding complex I^50^ and strain BW25113Δ*nuo*, Δ*ndh* lacks in addition *ndh* encoding for the alternative NADH-dehydrogenase (M. Vranas, D. Dekovič and T. Friedrich, unpublished results). BW25113∆*nuo* and BW25113∆*nuo,*∆*ndh* cells were transformed either with the parental plasmid pBAD*nuo*_*his*_ or the mutant plasmid pBAD*nuo*_*his*_ *nuoG* E617A expressing the entire complex I. Chloramphenicol (170 μg/mL), ampicillin (100 μg/mL) and kanamycin (50 μg/mL) were added where necessary.

### Nitrate reductase purification, Enzyme activity and protein quantification

The nitrate reductase-deficient JCB4023 strain was transformed with pNarGH_His6_ allowing expression of an inactive and Moco-less His-tagged NarGH complex (i.e. apoNarGH) while pNarGH_His6_J allows for expression of a fully active His-tagged NarGH complex (i.e. holoNarGH). Cells were grown aerobically at 37 °C in Terrific Broth medium and enzyme overproduction was performed using 0.2 mM isopropyl 1-thio-β-D-galactopyranoside. Holo and apoNarGH purification were performed by Ni-affinity chromatography as described^9^ and the purified proteins in Tris-HCl 40 mM pH 7.6, 8% glycerol were fast-frozen in liquid nitrogen prior conservation at −80 °C. Nitrate reductase activity was measured with standard assays using reduced benzyl viologen as electron donor^51^ and enzyme concentration was estimated by rocket immunoelectrophoresis as previously described using antibodies raised against NarGH^9^.

### Size exclusion chromatography

A Superose 6 10/300 GL column (GE Healthcare) was equilibrated with 10 CV of Tris-HCl 40 mM pH 7.6, 8% glycerol buffer using an ÄKTA purifier fast-protein liquid chromatography (FPLC) machine (GE Healthcare). Molecular weight calibration of the column was done by using the following standard markers as recommended by the manufacturer (Sigma-Aldrich): blue dextran (2000 kDa), thyroglobulin (660 kDa), apoferritin (443 kDa), β-amylase (200 kDa) and alcohol dehydrogenase (150 kda). Holo, apo and R108A NarGH complexes were injected at a flow rate of 0.3 ml/min, protein elution being monitored at 280 nm. Varying protein concentration did not influence the elution profile.

### Metal analysis

Metal analysis of purified nitrate reductase (holoNarGH and NarG_R108A_H) was performed using inductively coupled plasma mass spectrometry (ICP-MS). Protein samples were wet-washed in a 1:5 mixture with 65% v/v nitric acid, for two days. Samples were then diluted with 3 mL of ultrapure water 18 MΩ. The solutions obtained were then analysed in triplicate by ICP-MS using a Thermo Series II ICP/MS apparatus (Thermo-Electron, Les Ulis, France). As reference, a multi-element standard solution (SCP Sciences, Canada) was used. Metal concentrations were finally determined using Plasmalab software (Thermo-Electron, Les Ulis, France).

### EPR spectroscopy

X-band EPR spectra were recorded using a Bruker-Biospin EleXsys E500 spectrometer equipped with a standard rectangular Bruker EPR cavity fitted to an Oxford Instruments helium flow cryostat. Redox poised samples were prepared as previously described^9^,^52^. Redox potentials are given in the text with respect to the standard hydrogen electrode.

### Two-hybrid Assays

Protein interactions have been detected using a bacterial two-hybrid approach based on functional reconstitution of adenylate cyclase activity as previously reported^29^,^53^. A full-length NarG protein and a shortened version devoid of the first 41 residues (known to constitute a distinct NarJ binding site not involved in cofactor insertion^10^) were used as prey while tested for interaction against NarJ and the Zip domain used a negative control. Protein interactions were estimated by β-galactosidase activity measurements in cells at mid-log phase grown at 30 °C in L-broth medium supplemented with 0.5 mM isopropyl-1-thio-β-D-galactopyranoside as described previously^29^.

### Complex I production, enzyme activity and stability analysis

Cells were grown aerobically in a 10 L fermenter or as 400 mL cultures in 1 L baffled conical flasks in autoinduction medium. The medium contains 0.5% (w/v) yeast extract, 1.0% (w/v) peptone, 25 mM Na_2_HPO_4_, 25 mM KH_2_PO_4_, 50 mM NH_4_Cl, 5 mM Na_2_SO_4_, 2 mM MgSO_4_, 0.5% (w/v) mannitol, 0.05% (w/v) glucose, 0.2% (w/v) l-arabinose, 50 mg/L riboflavin, 30 mg/L ferric ammonium citrate and 0.5 mM l-cysteine. Cells were harvested before entering the stationary phase. Complex I-enriched cytoplasmic membranes were isolated as described in ref. 27. The NADH oxidase activity and the NADH/ferricyanide oxidoreductase activity were measured as already described^54^,^55^. Protein concentrations were determined according to the biuret method. The overproduction of the variant and complex I was verified by western-blot using antibodies raised against Tetra-His (Qiagen) on samples run on 10% SDS-polyacrylamide gels. The stability of the NuoG_E617A_ complex I variant was determined by sucrose gradient density centrifugation as described^27^.

### SAXS experiments

SAXS data on size-exclusion-chromatography-purified proteins were collected at SWING beamline at the SOLEIL Synchrotron. The sample-to-detector distance was set at 1821 mm for holoNarGH and apoNarGH, and at 1845 mm for NarG_R108A_H, with wavelength λ = 1.0 Å. These setups gave access to scattering vectors q ranging respectively from 0.008 to 0.48 Å^−1^, and from 0.006 to 0.5 Å^−1^, where q = 4πsinθ/λ, and 2θ is the scattering angle. The protein samples (~25 mg/mL in Tris-HCl 40 mM pH 7.6, 8% glycerol) were eluted on an online HPLC device with an Agilent BioSEC-3 300 Å column upstream the measurement capillary^56^ to separate putative aggregates and 200 frames of 1 sec were recorded during protein elution. The background buffer signal (100 frames of 1 sec) was recorded in the first minutes of the elution, before any elution peak. Importantly, no significant difference was observed in the SAXS spectra of all three protein samples in the initial fractions of the elution peak indicating the presence of homogeneous samples. Subsequently, those frames exhibiting the same radius of gyration (R_g_) were averaged, and corrected from the buffer signal. Primary data treatment was made using the program package PRIMUS^57^. The R_g_s were derived from the Guinier approximation (qR_g_ < 1.0)^58^. The distance distribution function P(r) and the maximum diameter D_max_, were determined using GNOM^59^. Molecular weights were assessed directly from the scattering curves by SAXSMoW (SAXS Molecular Weight)^14^ (http://www.if.sc.usp.br/~saxs/). The inferred oligomeric state of holoNarGH and variants in solution, confirmed by determination of the volume of the protein by DAMMIF, was found to be different from the X-ray crystal structure. SAXS can indeed reveal different solution behavior compared to the crystalline assembly, probably because of crystal packing or altered intermolecular interactions due to the high solute concentrations in the crystallization solution^60^. *Ab initio* shape determination was performed using DAMMIF^61^. Fifteen dummy-atoms models were generated without symmetry constraints, and averaged using the program suite DAMAVER^62^.

### Rigid body modelling

Rigid body modeling was performed with SASREF without any symmetry constraint^63^ to compare atomic models deduced from X-ray crystal structures to the structures in solution. The atomic coordinates of the NarGH heterodimer were taken from the X-ray crystal structure (Protein data bank code 1q16). Models for SAXS comparison were obtained by applying manually several modifications on the above-mentioned atomic structure using PyMOL^64^. For instance, evaluation of the impact of the disruption of the solvent-exposed salt bridge between Arg108 and Glu794 in NarG on the SAXS profile has been done by modification of two loops (Tyr105-Tyr111 and Ser811-Ser822) giving rise to a new atomic model used as template for SASREF. The same holds true for questioning the mobility of domain IV of NarG or alternative positions of the NarG subunit. For each condition, SASREF was run 10 times with three same initial atomic structures centered at the origin, without symmetry constraints. Finally, we used DAMSEL^62^ to select the best representative structures. The quality of fit between models and experimental SAXS data are usually assessed by the χ value, which is a global parameter, and in addition, suffers from over-fitting and a low sensitivity in case of noisy data sets. The quality of fit between models and experimental SAXS data was therefore also assessed by plotting the residuals, which presents the advantage of allowing a fine analysis of the fitting quality at all points of the scattering profile.

### Site-directed Mutagenesis

The oligonucleotides used in this study are described in Supplementary Table 2. Introduction of the R108A substitution in NarG was performed in two steps. The pNarGH_His6_J plasmid was PCR-amplified using the primer pair *narG* R108A_fwd and *narG* R108A_rev (Supplementary Table 2). After treatment with *Dpn*I, electrocompetent DH5α cells were transformed with the PCR product and screened for ampicillin resistance. The substitution was then verified by sequencing of the *narG* ORF. To avoid any PCR-induced error in the rest of the plasmid, the mutated plasmid was restricted using *EcoR*I allowing the isolation of a fragment containing the mutation. The restricted fragment was then reintroduced into pNarGH_His6_J to yield the pNarG_R108A_H_His6_J plasmid allowing overproduction of the NarG_R108A_H_His6_ complex. A similar strategy was employed for introduction of the R108A substitution in the pT25-NarG and pT25-NarG(Δ1–41) plasmids and sequence verified.

Introduction of the E617A substitution in NuoG was performed in several steps. At first, the mutation was introduced in the pUC*nuoE-G* plasmid using the primer pair *nuoG* E617A_fwd and *nuoG* E617A_rev (Supplementary Table 2) to create the plasmid pUC*nuoE-G* E617A. The mutation was subsequently transferred to the pBAD*nuo*_*his*_ plasmid by λ-Red-mediated recombination as followed. Electrocompetent DH5αΔ*nuo*/pKD46 cells were prepared and electroporated. The *nptI-sacB* cartridge was amplified from pVO1100 by PCR with the primer pair *nuoG::nptI-sacB_*fwd and *nuoG::nptI-sacB_*rev (Supplementary Table 2). To integrate the cartridge into pBAD*nuo*_*his*_ by λ-Red-mediated recombination^65^, electrocompetent DH5αΔ*nuo*/pKD46 cells were mixed with 50 ng of pBAD*nuo*_*his*_ and 400 ng of the PCR product. Recombinants were selected on LB-agar supplemented with kanamycin. Plasmids were isolated from Km^R^ clones and purified by transformation of DH5α and growth on LB-agar supplemented with kanamycin. The *nptI-sacB* cartridge on pBAD*nuo*_*his*_ was replaced by the PCR product containing the mutation by recombination. A linear dsDNA was amplified from pUC*nuoE-G* E617A by PCR with the primer pair *nuoG*_beg__fwd and *nuoG*_rec__rev (Supplementary Table 2). Electrocompetent DH5αΔ*nuo*/pKD46 cells were co-transformed with 50 ng pBAD*nuo*_*his*_ *nuoG::nptI-sacRB* and 200 ng of PCR product. Recombinants were selected on YP-agar (1% w/v peptone, 0.5% yeast extract) supplemented with chloramphenicol and 10% (w/v) sucrose at 30 °C. Plasmids from Cm^R^ and Suc^R^ clones were isolated. The mutation was confirmed by DNA sequencing.

### Bioinformatics

Open reading frames (ORFs) coding for catalytic subunits homologous to Nar, Psr, Dms, Dor, Tor, Nap, Fdh, NuoG and Aio were retrieved from the National Center for Biotechnology Information (http://www.ncbi.nlm.nih.gov) using the NarG sequence from *E. coli,* PsrA sequence from *Wolinella succinogenes,* DmsA from *E. coli,* DorA from *Rhodobacter sphaeroides,* NapA from *Rhodobacter sphaeroides,* FdhF and FdnG from *E. coli,* NuoG from *E. coli* and AioA from NT-26, as query templates in BLAST searches. Structures were obtained from the pdb database (http://www.rcsb.org/pdb/welcome.do). Structural alignments were obtained using the root-mean-square fit option of the Swiss-Pdb Viewer (version 3.7; http://www.expasy.ch/spdbv). Multiple sequence alignments of recognized subfamilies of Mo-*bis*PGD subunits were automatically produced using ClustalX^66^ and T-Coffee^67^. The automatically generated alignments were subsequently refined using Seaview^68^ with respect to functionally conserved residues and crystal structures. Phylogenetic trees were reconstructed from these alignments using the Neighbor-Joining (NJ)-algorithm implemented in ClustalX or MEGA5.

## Additional Information

**How to cite this article**: Arias-Cartin, R. *et al.* Redox cofactors insertion in prokaryotic molybdoenzymes occurs via a conserved folding mechanism. *Sci. Rep.* **6**, 37743; doi: 10.1038/srep37743 (2016).

**Publisher's note:** Springer Nature remains neutral with regard to jurisdictional claims in published maps and institutional affiliations.

## Supplementary Material

Supplementary Information
