# Supplementary material for: Redox cofactors insertion in prokaryotic molybdoenzymes occurs via a conserved folding mechanism
Source: Sci Rep. 2016 Nov 25;6:37743. doi: 10.1038/srep37743 (PMC5123574; doi:10.1038/srep37743)
Supplement: Supplementary Information [file srep37743-s1.pdf]

Redox cofactors insertion in prokaryotic molybdoenzymes occurs via a conserved folding mechanism

Rodrigo Arias-Cartin[1‖], Pierre Ceccaldi[1,2§], Barbara Schoepp-Cothenet[2], Klaudia Frick[4], Jean-Michel Blanc[1‡], Bruno Guigliarelli[2], Anne Walburger[1], Stéphane Grimaldi[2], Thorsten Friedrich[4], Véronique Receveur-Brechot[3*], Axel Magalon[1*]

[1]Aix-Marseille Univ, CNRS, IMM, LCB UMR7283, Marseille, France & [2]Aix-Marseille Univ, CNRS, IMM, BIP UMR7281, Marseille, France. [3]Aix-Marseille Univ, CNRS, INSERM, Institut Paoli-Calmettes, CRCM UMR7258, Marseille, France. [4]Institut für Biochemie, Albert-Ludwigs-Universität, Freiburg, Germany.

‖**Present address:** Microbial Sciences Institute, Yale University, Department of Molecular, Cellular and Developmental Biology, West Haven, CT 06516, USA

§**Present address**: Chemistry Department, Boston University, 590 Commonwealth Avenue, 02215 Boston, MA, USA

‡**Present address:** Plateforme de Biochimie et Biophysique des protéines, SFR Bordeaux Neurocampus, Neurocentre Magendie, 33077 Bordeaux, France

Supplementary Figure 1: Size exclusion chromatography of all forms of NarGH. Gel filtration of holoNarGH (black), apoNarGH (red) and $NarG_{R108A}H$ (green) was performed on a Superose 6 10/300 GL column (GE Healthcare) equilibrated with Tris-HCl 40 mM pH 7.6 glycerol 8% buffer at a flow rate of 0.3 ml/min. HoloNarGH elutes as a main peak at 584 kDa (trimer), apoNarGH and $NarG_{R108A}H$ elute as a broad peak reflecting an equilibrium between trimers and dimers (444 kDa). An additional peak eluting at ~ 15 ml and corresponding to an apparent Mw of 148 kDa may correspond to a monomeric species in both the apo and variant samples but absent in the holoNarGH sample. The molecular weight (Mw) of the peaks was determined from the plot shown in the inset where Ve is the elution volume of each protein and Vo is the void volume of the column as determined using blue dextran. Molecular weight calibration of the column was done by using standard markers (Sigma-Aldrich) (see methods). The grey shaded zone highlights the eluted fractions considered for SAXS experiments.

Supplementary Figure 2: Representative arrangement of the five domains of NarG. The NarGH structure (issued from pdb 1Q16) is represented in cartoon. NarG is subdivided into domain I (green), domain II, (orange), domain III (magenta), domain IV (dark blue) and domain V (cyan). NarH is colored in gray. Metal centers are shown in spheres within the NarGH complex.

Supplementary Figure 3: A conserved surface-exposed salt bridge is present on NarG and NuoG. The cartoon models are colored by their secondary structure elements. A) shows NarG with the Mo-*bis*PGD cofactor and the FeS cluster FS0 (pdb entry: 1Q16) [8]. B) shows NuoG with the FeS cluster N7 (pdb entry: 3IAM) [65]. C) shows a superposition of the structure between NarG (colored by its secondary structure elements in red and green) and NuoG (colored in grey) with a RMSD of 2.962Å. This view depicts the overall conservation of the secondary structure elements surrounding the flexible loops maintained by the salt bridge. The

bridge building amino acids and the metal cofactors are highlighted, labelled and represented in stick.

Supplementary Figure 4: Phylogenetic analysis supports conservation of the structural domain of Mo-bis-PGD enzymes. 3D structure-based NJ-phylogenetic tree of the Mo-bis-PGD-enzymes respiratory nitrate reductase (Nar), periplasmic nitrate reductase (Nap), DMSO/TMAO reductase (Dms/Dor/Tor), formate dehydrogenase (Fdh), arsenite oxidase (Aio) and polysulfide reductase (Psr) together with close related NuoG subunit from complex I. Violet and orange denote eury- and cren-archaeal branches, dark green, cyan and light green stand for proteobacteria, firmicutes and other bacteria, respectively. Open and closed dots indicate bootstrap values for the deep branching exceeding 70 and 90%, respectively.

Supplementary Figure 5: Immunoblot analysis of complex I integrity. Membranes from strain BW25113Δ*ndh*Δ*nuo*/pBAD*nuo* expressing the entire complex I with his-tagged NuoF and containing the $NuoG_{E615A}$ variant (1) and from strain BW25113Δ*ndh*Δ*nuo*/pBAD*nuo* expressing the wild-type complex I with his-tagged NuoF (2). The apparent molecular masses of the standard Page Ruler Prestained Protein Ladder in kDa are indicated (lane MM). Lanes 1 and 2 were loaded with 100 µg protein. A single band corresponding to NuoF detected by an anti-His antibody (Qiagen) is observed in lanes 1 and 2. Quantitative analysis by an Imager system revealed a 40% reduced signal in lane (1) as compared to lane (2).

Supplementary Table 1. Bacterial strains and plasmids used in this study

Strain/Plasmid	Relevant genotype/Description	Reference
JCB4023	RK4353, Δ*napA-B, narG*::*ery*, Δ*narZ*::Ω, Spc^R	[66]
DH5αΔ*nuo*	DH5α, Δ*nuo*	[41]
BW25113Δ*nuo*	BW25113Δ*nuo*	[46]
BW25113 Δ*nuo*Δ*ndh*	BW25113 *Δnuo, Δndh*	(Vranas M, Dekovic D and Friedrich T, unpublished results)
pT18-Zip	pT18, Leucine zipper fused to T18 fragment (225–399 amino acids of CyaA), Amp^R	[46]
pT18-NarJ	pT18, NarJ-T18 fusion protein, Amp^R	[46]
pT25-NarG	pT25, T25-NarG fusion protein, Cm^R	[46]
pT25-NarG$_{R108A}$	pT25, T25-NarG$_{R108A}$ fusion protein, Cm^R	This work
pT25-NarG(Δ1-41)	pT25, T25-NarG(41-1247) fusion protein, Cm^R	[46]
pT25-NarG$_{R108A}$(Δ1-41)	pT25, T25-NarG$_{R108A}$(41-1247) fusion protein, Cm^R	This work
pNarGH$_{His6}$	pJF119EH, P$_{tac}$-(*narGH$_{His6}$*), Amp^R	[9]
pNarGH$_{His6}$J	pJF119EH, P$_{tac}$-(*narGH$_{His6}$J*), Amp^R	[9]
pNarG$_{R108A}$H$_{His6}$J	pJF119EH, P$_{tac}$-(*narG$_{R108A}$H$_{His6}$J*), Amp^R	This work
pBAD*nuo$_{his}$*	pBAD expressing entire Nuo complex with a his-tagged NuoF subunit	[46]
pBAD*nuo$_{his}$,G$_{E617A}$*	As pBAD*nuo$_{his}$* but expressing the NuoG$_{E615A}$ variant	This work
pUC*nuoE-G*	pUC, *nuoE-G*, Amp^R	(Kohlstädt M and Friedrich T, unpublished results)
pUC*nuoE-G$_{E617A}$*	pUC, *nuoE-G$_{E615A}$*, Amp^R	This work
pKD46	λ red recombinase, Amp^R	[45]
pV01100	Amp^R, kan^R, nptI-sacR, sacB	[51]

Supplementary Table 2: DNA-oligonucleotides used throughout this work

Oligonucleotide	Sequence
narG R108A_fwd[1]	5'-CGCGGTGCCAGCTACTCCTGGTATCTTTACAGTGCCAAC**GCC**CTG-3'
narG R108A_rev[1]	5'-CATTTTCATCAGGCGTTTGCGCATCATCGGGTATTTCAGG**GCG**TTG-3'
nuoG E617A_fwd[2]	5'-CTGCCAGCTTTGCT***GCT***AGCGACGGTACGGTGATC-3'
nuoG E617A_rev[2]	5'-GATCACCGTACCGTCGCT***AGC***AGCAAAGCTGGCAG-3'
*nuoG::nptI-sacB*_fwd[3]	5'-GAATACGAGGTCAACGGAGCGGACAACCTGCTGGAAGCTT **CACGTTGTGTCTCAAAATCTCTGATG**-3'
*nuoG::npt-sacB*_rev[3]	5'-TCAACCGGCAGCGTGACCGTGTTGCCATCGTAACTAAAGG AGACGCG**GAATTCCCCGGGGGATCCG**-3'
nuoG$_{beg}$_fwd[4]	5'-CGATTAACGCTCAGTCTC-3'
nuoG$_{rec}$_rev[4]	5'-GTGCCTCCTTGAGATCCTC-3'

[1]This primer pair was used to introduce a point mutation in *narG*. The modified codon is printed bold.

[2]This primer pair was used to introduce a point mutation in *nuoG*. The modified codon is printed bold, silent mutations to insert a new NheI restriction sites are marked italic and the newly introduced restriction sites is underlined.

[3]This primer pair was used for the insertion of the *nptI-sacB* cartridge. Regions homologous to *nuoG* are underlined. Regions homologous to the *nptI-sacB* cartridge are marked bold.

[4]This primer pair was used for the amplification of the PCR fragment carrying the point mutation.

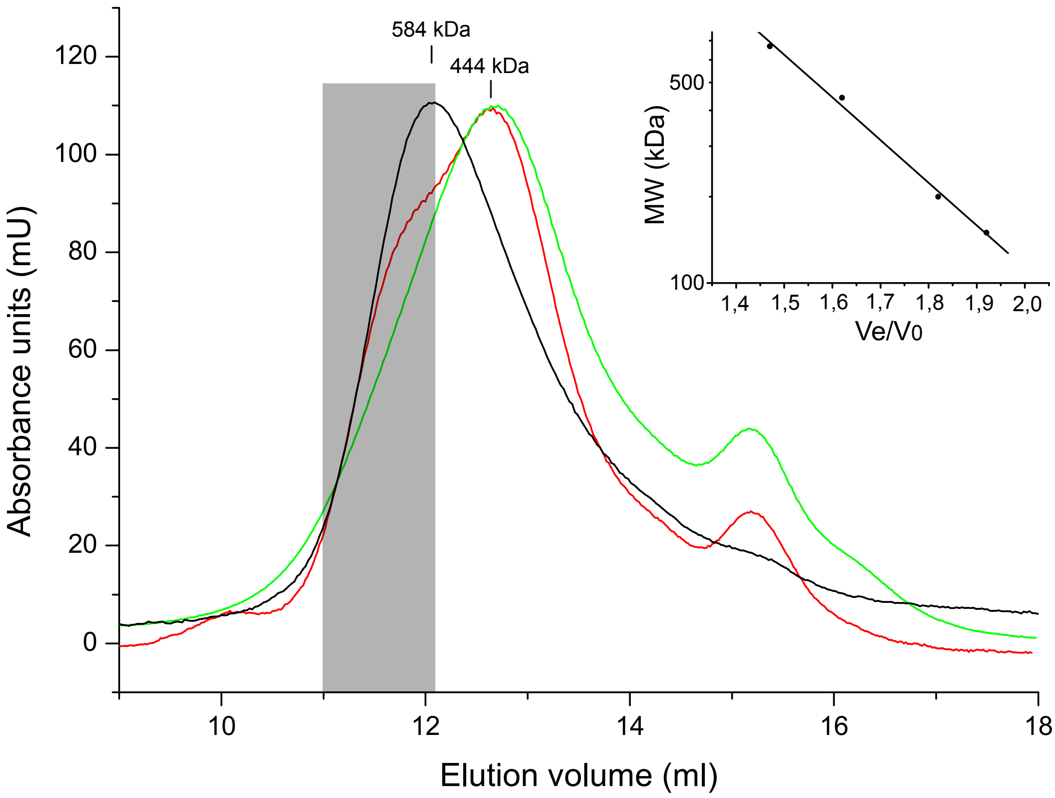

584 kDa
444 kDa
Absorbance units (mU)
Elution volume (ml)
MW (kDa)
Ve/V0

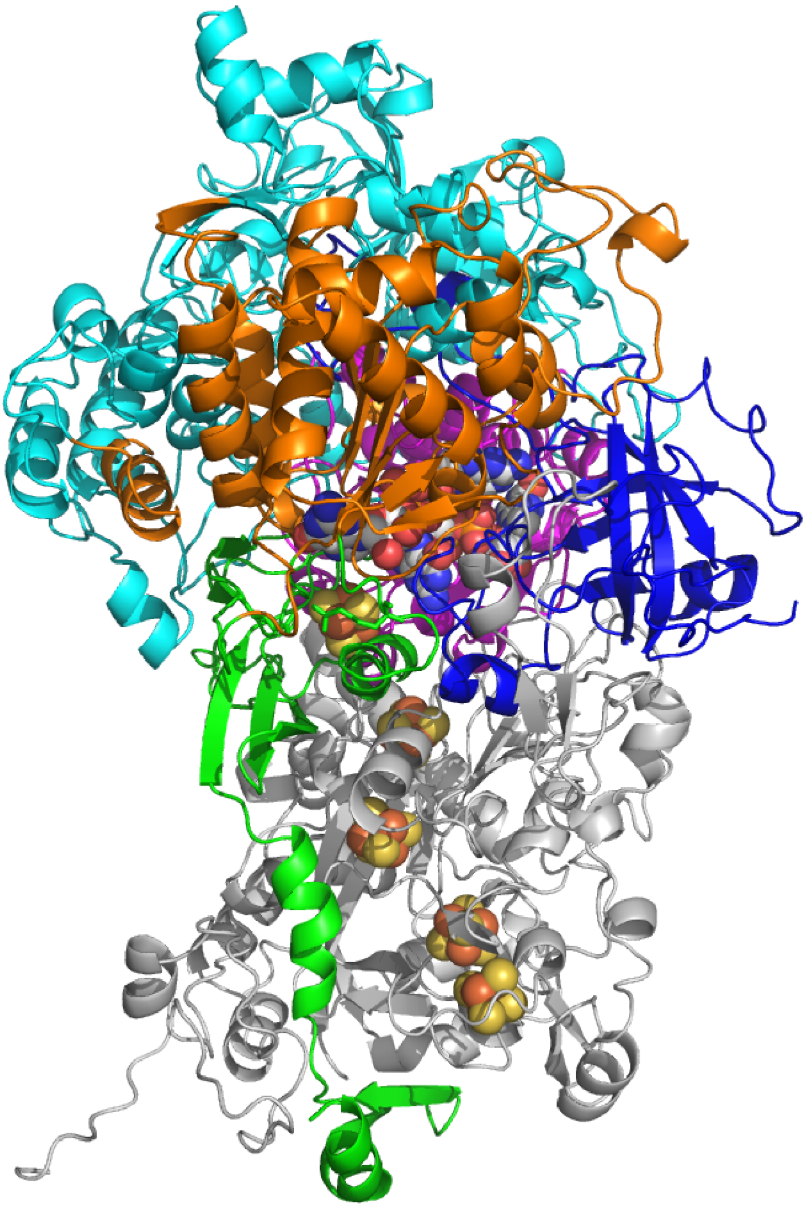

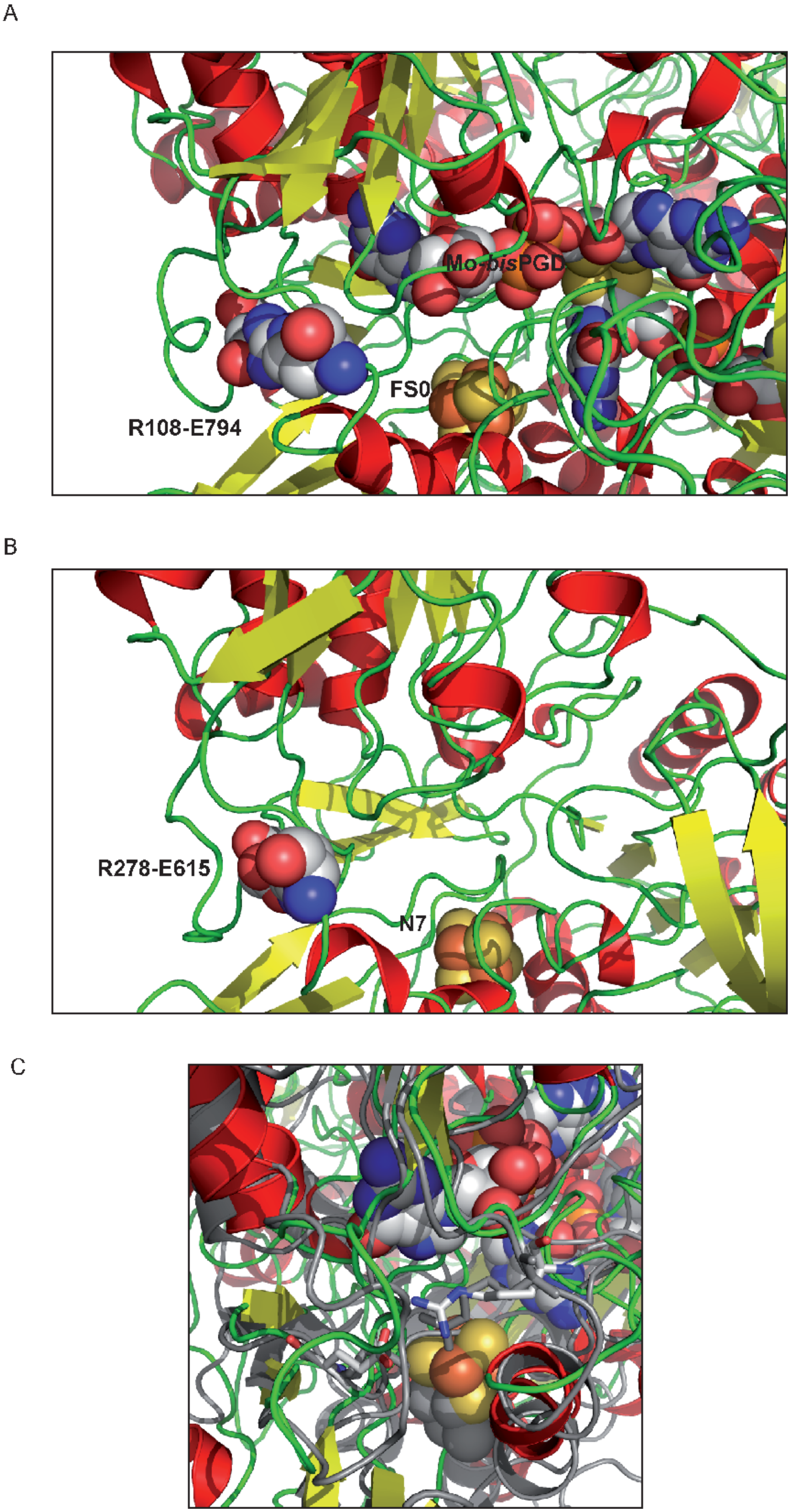
A
Mo-*bis*PGD
FS0
R108-E794
B
R278-E615
N7
C

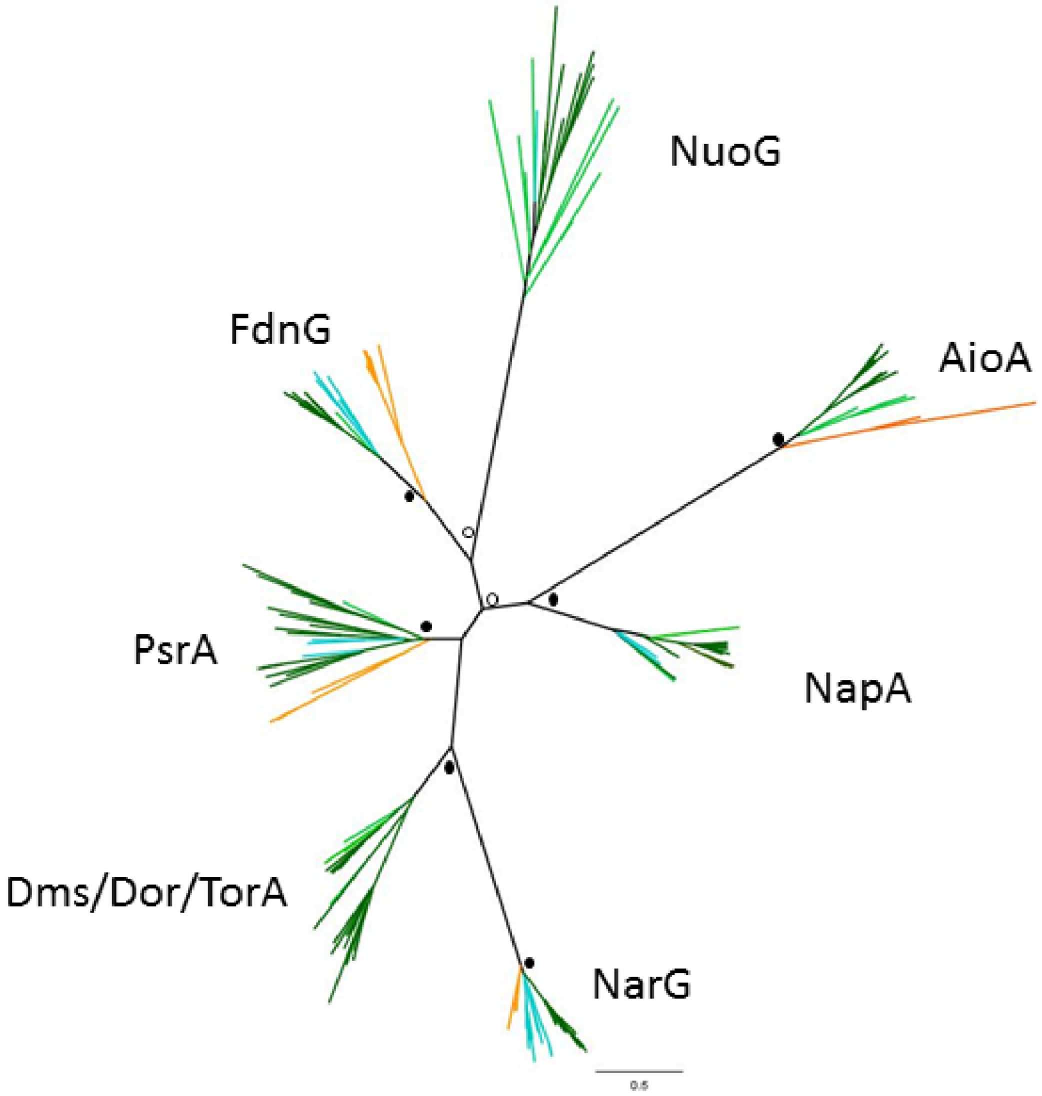
NuoG
FdnG
AioA
PsrA
NapA
Dms/Dor/TorA
NarG
0.5

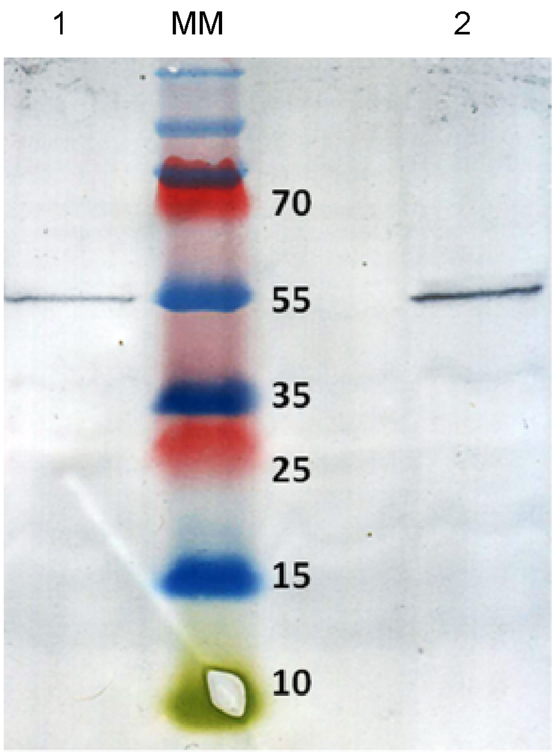

1
MM
2
70
55
35
25
15
10
